# Combined genomic and proteomic approaches reveal DNA binding sites and interaction partners of TBX2 in the developing lung

**DOI:** 10.1186/s12931-021-01679-y

**Published:** 2021-03-17

**Authors:** Timo H. Lüdtke, Irina Wojahn, Marc-Jens Kleppa, Jasper Schierstaedt, Vincent M. Christoffels, Patrick Künzler, Andreas Kispert

**Affiliations:** 1 Institut Für Molekularbiologie, Medizinische Hochschule Hannover, Hannover, Germany; 2 Present Address: Plant-Microbe Systems, Leibniz Institute of Vegetable and Ornamental Crops, Großbeeren, Germany; 3 Department of Anatomy, Embryology and Physiology, Academic Medical Center, University of Amsterdam, Amsterdam, The Netherlands; 4 Institut Für Pflanzengenetik, Leibniz Universität Hannover, Hannover, Germany

**Keywords:** Tbx2, Pulmonary mesenchyme, Lung development, NuRD, HDAC, CBX3, HMGB2, PBX1

## Abstract

**Background:**

*Tbx2* encodes a transcriptional repressor implicated in the development of numerous organs in mouse. During lung development TBX2 maintains the proliferation of mesenchymal progenitors, and hence, epithelial proliferation and branching morphogenesis. The pro-proliferative function was traced to direct repression of the cell-cycle inhibitor genes *Cdkn1a* and *Cdkn1b*, as well as of genes encoding WNT antagonists, *Frzb* and *Shisa3,* to increase pro-proliferative WNT signaling. Despite these important molecular insights, we still lack knowledge of the DNA occupancy of TBX2 in the genome, and of the protein interaction partners involved in transcriptional repression of target genes.

**Methods:**

We used chromatin immunoprecipitation (ChIP)-sequencing and expression analyses to identify genomic DNA-binding sites and transcription units directly regulated by TBX2 in the developing lung. Moreover, we purified TBX2 containing protein complexes from embryonic lung tissue and identified potential interaction partners by subsequent liquid chromatography/mass spectrometry. The interaction with candidate proteins was validated by immunofluorescence, proximity ligation and individual co-immunoprecipitation analyses.

**Results:**

We identified *Il33* and *Ccn4* as additional direct target genes of TBX2 in the pulmonary mesenchyme. Analyzing TBX2 occupancy data unveiled the enrichment of five consensus sequences, three of which match T-box binding elements. The remaining two correspond to a high mobility group (HMG)-box and a homeobox consensus sequence motif. We found and validated binding of TBX2 to the HMG-box transcription factor HMGB2 and the homeobox transcription factor PBX1, to the heterochromatin protein CBX3, and to various members of the nucleosome remodeling and deacetylase (NuRD) chromatin remodeling complex including HDAC1, HDAC2 and CHD4.

**Conclusion:**

Our data suggest that TBX2 interacts with homeobox and HMG-box transcription factors as well as with the NuRD chromatin remodeling complex to repress transcription of anti-proliferative genes in the pulmonary mesenchyme.

**Supplementary Information:**

The online version contains supplementary material available at 10.1186/s12931-021-01679-y.

## Background

In the mammalian lung, trachea, bronchi and bronchioles form a tree-like system of tubes that conduct the air to thin-walled terminal sacs, the alveoli, where the exchange of carbon dioxide and oxygen occurs. This elaborate epithelial system arises from a simple outgrowth of the foregut endoderm by a complex program of specification, proliferative expansion, branching morphogenesis, proximal–distal patterning and differentiation during embryonic development [1]. All of these epithelial processes depend on cues from surrounding mesenchymal cells and the visceral pleura, the mesothelial lining of the lung. Branching morphogenesis occurs mostly during the pseudoglandular stage of lung development which extents in mice from embryonic day (E)12.5 to E16.5. Here, the pulmonary mesenchyme acts as a source for signals that direct the proliferative expansion and branching of the distal epithelial tips of the developing airways. In turn, endodermal and mesothelial signals maintain a proliferative undifferentiated state of the pulmonary mesenchyme, thus, preventing its differentiation into chondrocytes, smooth muscle cells (SMCs) and various types of fibroblasts that will later ensheath the epithelial components of the mature lung [2, 3]. The cross-talk between all three pulmonary tissue compartments is executed by a number of different signaling molecules including SHH, BMPs, FGFs and WNTs [4–9].

Orchestration and interpretation of these reciprocal signaling cascades require the activity of transcription factors that regulate the signals and their activities in time and space but also impinge onto the cell-cycle machinery to assure the pro-proliferative undifferentiated state in either tissue compartment. T-box proteins are members of a large, evolutionary conserved family of transcriptional regulators that share a highly conserved DNA-binding region, namely the T-box [10]. Transcriptional regulation by T-box proteins underlies a multitude of cellular processes including proliferation and differentiation in diverse contexts of germ layer, tissue and organ development as evidenced by severe embryonic defects in men and animals with loss- and gain-of-function of these genes [11, 12].

Our previous work characterized the T-box transcription factor TBX2 as a mesenchymal regulatory hub during lung development. *Tbx2* and the closely related *Tbx3* gene are predominantly expressed in mesenchymal precursors that surround the distal endodermal tips. The expression largely depends on epithelial SHH signals with modulatory input from epithelial BMP4, mesenchymal TGFs, and WNTs possibly emerging from both compartments [13, 14]. Loss of *Tbx2* and even more, the combined loss of *Tbx2* and *Tbx3* in mice, results in arrest of mesenchymal proliferation, premature mesenchymal differentiation and an arrest of epithelial branching morphogenesis leading to lung hypoplasia at birth. Prolongation of TBX2 expression into adulthood leads to hyperproliferation and maintenance of mesenchymal progenitor cells. These cellular changes were traced to a molecular function of TBX2 to directly repress expression of the cell-cycle inhibitor genes *Cdkn1a* and *Cdkn1b*, as well as of genes encoding WNT antagonists, *Frzb* and *Shisa3,* which in turn increases pro-proliferative WNT signaling [13, 15].

Despite these important molecular insights, we still lack a survey of all direct target genes of TBX2 in the mesenchyme of the developing lung and of the nature and configuration of DNA-binding sites present in these genes. Moreover, we do not know with which other transcription factors, corepressors and chromatin remodeling complexes TBX2 interacts to achieve target gene specificity and repression in this developmental context.

Here, we set out to experimentally address these questions. Using a combination of transcriptional profiling by microarrays and ChIP-Seq technology, we identified additional targets of TBX2 activity including *Ccn4* and *Il33*, and describe the consensus binding site of TBX2 in the developing lung. Additionally, we identified and characterized protein binding partners of TBX2 that may aid in specific repression of these target genes.

## Methods

### Mouse strains and genotyping

All mouse strains used in this study: *Tbx2*^*tm1.1(cre)Vmc*^ (synonym: *Tbx2*^*cre*^) [16], *Tbx2*^*tm2.2Vmc*^ (synonym: *Tbx2*^*fl*^) [17], *Gt(ROSA)26*^*Sortm4(ACTB−tdTomato,−EGFP)Luo/J*^ (synonym: *R26*^*mTmG*^) [18] were maintained on an NMRI outbred background. Embryos for analyses were obtained from matings of NMRI wildtype mice, and from matings of *Tbx2*^*cre/*+^ males with *R26*^*mTmG/mTmG*^;*Tbx2*^*fl/fl*^ or *Tbx2*^*cre/*+^ females. To time the pregnancy, vaginal plugs were checked on the morning after mating and noon was taken as embryonic day (E) 0.5. On the day of harvest, pregnant females were sacrificed by cervical dislocation. Embryos and lungs were dissected in PBS. For both in situ hybridization and immunofluorescence analyses, embryos were fixed in 4% PFA/PBS, transferred to methanol and stored at − 20 °C. PCR genotyping was performed on genomic DNA prepared from ear clips of adult mice or from embryonic tissues.

All animal work conducted for this study was approved by the local authorities (Niedersächsisches Landesamt für Verbraucherschutz und Lebensmittelsicherheit; permit number AZ33.12-42502-04-13/1356) and was performed at the central animal laboratory of the Medizinische Hochschule Hannover in accordance with the National Institute of Health guidelines for the care and use of laboratory animals.

### Chromatin immunoprecipitation DNA-sequencing (ChIP-seq) assays

For ChIP-Seq analysis, a total of 100 E14.5 wildtype lungs were minced in PBS into pieces of 100–500 µm. The tissue was incubated in 1.6% formaldehyde/PBS for 20 min before glycine was added to a final concentration of 1% and incubation continued for 10 min at room temperature. After a washing step with PBS, the tissue was stored at − 80 °C until further use. ChIP reactions were performed with the SimpleChIP® Plus Enzymatic Chromatin IP Kit (Magnetic Beads) (#9005, Cell Signaling Technology, Danvers, MA, USA) following manufacturer’s instructions. Nuclease treatment for fragmentation of chromatin was prolonged to 30 min and nuclease concentration was doubled to obtain fragments of 300 bp in average. The DNA-containing supernatants were incubated with a ChIP grade anti-TBX2 antibody (1:50; sc-514291 X, Santa Cruz Biotechnology Inc., Santa Cruz, CA, USA), anti-Histone H3 (1:50; #9005, Cell Signaling Technology) or an IgG control (1:50; #9005, Cell Signaling Technology) for 1 h at room temperature, and together with ChIP-Grade ProteinG Magnetic Beads (#9006S, Cell Signaling Technology) overnight at 4 °C.

The DNA precipitates were passed to the Research Core Unit Genomics of Hannover Medical School. Library preparation was performed with NEBNext® Ultra™ II DNA Library Prep Kit for Illumina® (E7645S, New England Biolabs, Ipswich, MA, USA) and next generation sequencing was performed on Illumina NextSeq High Output 500/550 flow cells with a reading depth of 15 million 75 bp paired-end reads (FC-404-2005, Illumina, San Diego, CA, USA) using NEBNext® Multiplex Oligos for Illumina® (96 Unique Dual Index Primer Pairs) (E6440S, New England Biolabs,) following manufacturer’s instructions. ChIP peaks were mapped against the GRC38/mm10 genome (NCBI BioProject Accession: PRJNA20689) using MACS2 callpeak integrated in Galaxy version 2.1.1.20160309.1 [19]. ChIP peaks were visualized and manually analyzed using IGV software v.2.5.3 [20, 21]. Associated gene names were determined in Galaxy with “Fetch closest non-overlapping feature”, version 4.0.1. (https://usegalaxy.org). Gene ontology (GO) term analysis was performed with Genomic Regions Enrichment of Annotations Tool (GREAT, version 4.0.4, http://great.stanford.edu/public/html). De novo motif analysis on the data was performed with the FIMO tool in Galaxy (Version 4.11.1.0, https://usegalaxy.org) [22] for palindromic and non-palindromic sequences. For that purpose, sequence information from Macs2 callpeak data was gathered in Galaxy with the “Extract Genomic DNA” plugin (Version 2.2.3). Enriched motifs were compared to known transcription factor binding profiles with the TomTom Motif Comparison Tool version 5.1.1 (http://meme-suite.org/tools/tomtom) [23], using annotated sequences stored in Jaspar (http://jaspar.genereg.net) and footprintDB (http://floresta.eead.csic.es/footprintdb) databases.

### GO-term analysis of gene lists

Lists of gene symbols were imported into DAVID Bioinformatics Resources version 6.8 (https://david.ncifcrf.gov) [24] with annotations restricted to mouse. Gene lists imported into MouseMine websoftware (MGI 6.14) [25] were analyzed for ontology terms of biological processes determined with Holm–Bonferroni test correction and p-values smaller than 0.05.

### ChIP-PCR assays

Chromatin of ~ 20 wildtype and *Tbx2*-mutant lungs was isolated as described for ChIP-seq experiments and subjected to PCR amplification of gene-specific peak regions. Primers for a peak in *Ccn4*, chr15:66,883,385–66,883,657 were: 5′- CCAGAGAATGTCACACTCCAC-3′ and 5′- GCAGCTACTGGGTCTCTCA-3′. For peak #1 in *Il33 (*chr19:29,925,062–29,925,237): 5′-TGGTTCTCTGCCAAGTTCTG-3′ and 5′- TGCTCCACAGGTCCTAAGAT-3′; for peak #2 in *Il33 (*chr19:29,924,808–29,924,983): 5′- GGCTAAGGCAAGAAGATCATG-3′ and 5′-CCTGCCAATGTTACTGTTATC-3′.

### Proteomic analysis

Three independent proteomic analyses were performed using material of 100 E14.5 lungs each. The lung tissue was fixed and stored until further use as described for ChIP-seq assays. Tissue dissociation was achieved following the RIME protocol [26] utilizing a Minilys homogenizer (#P000673-MLYS0-A, Bertin Technologies, Montigny-le-Bretonneux, France) with mixed 1.4/2.8 mm ceramic beads (#91-PCS-CKM, VWR International, Radnor, PA, USA) and a sonification step of 3 × 20 pulses of an amplitude of 60% with a duty cycle of 75% (UP200H, Sonotrode S1, Ø1mm, Hielscher Ultrasonic GmbH, Teltow, Germany). Cell lysates were incubated overnight at 4 °C under constant rotation with ChIP-Grade ProteinG Magnetic Beads (#9006S, Cell Signaling Technology) conjugated either with normal rabbit IgG (#9005, Cell Signaling Technology) or ChIP grade mouse-anti-TBX2 antibody (1:50; sc-514291X, Santa Cruz). Enzymatic digestion and raw data processing steps were performed by the Research Core Unit Proteomics of the MHH. Liquid chromatography with subsequent tandem mass spectrometry (LC–MS/MS) was performed by the Department of Plant Proteomics of the Institute of Plant Genetics of the Leibniz-University Hannover. Extracted proteins were alkylated with iodacetamide and digested with trypsin overnight at 37 °C in 40 mM ammonium hydrocarbonate buffer containing 10% acetonitrile. The reaction was stopped by increasing the concentration of trifluoroacetic acid (TFA) to 5%. Samples were centrifuged at high speed and supernatants containing peptides were dried and stored at − 20 °C.

Apart from minor modifications, LC–MS/MS was performed as previously described [27]. Peptides were resuspended in 20 µl of 5% [v/v] acetonitrile and 0.1% [v/v] TFA, of which 1 µl were loaded onto a 2 cm C18 reversed phase trap column (Acclaim PepMap100, diameter: 100 µm, granulometry: 5 µm, pore size: 100 Å; Thermo Fisher Scientific, Waltham, MA, USA). Separation took place on a 50 cm C18 reversed phase analytical column (Acclaim PepMap100, diameter: 75 µm, granulometry: 3 µm, pore size: 100 Å; Thermo Fisher Scientific, Dreieich, Germany) using a 60 min non-linear 5–36% [v/v] acetonitrile gradient in 0.1% [v/v] formic acid for elution (250 nl/min; 33 °C). Eluting peptides were transferred into a Q-Exactive mass spectrometer (Thermo Fisher Scientific) by electrospray ionization (ESI) using a NSI source (Thermo Fisher Scientific) equipped with a stainless steel nano-bore emitter (Thermo Fisher Scientific). A spray voltage of 2.2 kV, capillary temperature of 275 °C, and S-lens RF level of 50% were selected. The data-dependent MS/MS run was conducted in positive ion mode using a top-10 method. MS1 spectra (resolution 70,000) and MS2 spectra (resolution 17,500) were recorded in profile mode from 20 to 100 min. Automatic gain control (AGC) targets for MS and MS/MS were set to 1E6 and 1E5, respectively. Only peptides with 2, 3, or 4 positive charges were considered. Raw data were processed using Max Quant (version 1.5, [28]), and Perseus software (version 1.6.2.3, [29]) and human and virus entries of Uniprot databases containing common contaminants. Proteins were stated identified by a false discovery rate of 0.01 on protein and peptide level and quantified by extracted ion chromatograms of all peptides.

Protein network analysis was performed using the STRING protein–protein interaction networks functional enrichment analysis tool v11 (https://string-db.org) [30] with MCL clustering with an inflation parameter of 2 as suggested by STRING, an interaction score of high confidence (0.700) and deactivating text mining as least meaningful interaction source.

### RNA in situ hybridization analysis

Non-radioactive in situ hybridization analysis of gene expression was performed on 10-μm paraffin sections of embryos using digoxigenin-labeled antisense riboprobes as described previously [31]. For each marker, sections from at least three mutant and control lungs were analyzed.

### Immunofluorescence

Detection of antigens was performed on 5-µm or 10-µm frontal sections through the lung region of paraffin-embedded embryos. Endogenous peroxidases were blocked by incubation in 6% H_2_O_2_ for 20 min. Antigen retrieval was achieved by citrate-based heat unmasking (H-3300, Vector Laboratories Inc., Burlingame, CA, USA). The following primary antibodies were used: anti-CBX3 (1:200; #PA5-30954, ThermoFisher Scientific, Waltham, MA, USA), anti-CHD4 (1:200; ab70469, Abcam plc, Cambridge, UK), anti-HDAC1 (1:200; #PA1-860, ThermoFisher Scientific), anti-HDAC2 (1:200; #51-5100, ThermoFisher Scientific), anti-HMGB2 (1:200; #ab124670, Abcam plc), anti-PBX1 (1:100; #PA5-82100, ThermoFisher Scientific), anti-TBX2 (1:200 or 1:2000; #07-318, Merck Millipore, Darmstadt, Germany), anti-TBX2 (1:200; #sc-514291X, Santa Cruz Biotechnology Inc.). Primary antibodies were detected by directly labeled fluorescence- or biotin-conjugated secondary antibodies (1:200; Invitrogen, Carlsbad, CA, USA; Dianova, Hamburg, Germany). The signal was amplified using a tyramide signal amplification (TSA) system (NEL702001KT, PerkinElmer, Waltham, MA, USA) according to the manufacturer’s instruction. Nuclei were stained with 4′,6-diamidino-2-phenylindole (DAPI, #6335.1, Carl Roth, Karlsruhe, Germany).

### In situ proximity ligation assay

Analysis of direct protein interactions on 10 µm frontal lung sections of E14.5 wildtype and *Tbx2*-mutant embryos was performed with the proximity ligation assay [32, 33] using the Duolink™ In Situ Red Starter Kit Mouse/Rabbit (DUO92101, Sigma Aldrich/Merck, Darmstadt, Germany) applying minor modifications of the manufacturer’s instructions. Antibody combinations were used and antibody retrieval was performed as described for immunofluorescence analysis. Sections were blocked with and primary antibody reaction was performed in blocking buffer from the tyramide signal amplification (TSA) system (NEL702001KT, PerkinElmer, Waltham, MA, USA) overnight at 4 °C, containing both corresponding primary antibodies for TBX2 and its putative interaction partner in a 1:100 dilution. After three washing steps with PBS/0.1% Tween20 for 5 min, the sections were blocked for 60 min with blocking buffer from the PLA kit and washed three times for 5 min in buffer A from the PLA kit before application of Duolink® PLA probes. Polymerase amplification reaction was performed for 150 min at 37 °C.

### Cell culture, co-transfections and co-immunoprecipitations

HEK293 cells (ACC 305, DSMZ, Braunschweig, Germany) were cultured in DMEM medium with GlutaMax™ (#61965-059, ThermoFisher Scientific) containing 10% FCS (#F2442, Merck), 100 units/ml penicillin, 100 µg/ml streptomycin (#15140122, ThermoFisher Scientific), 5% sodium pyruvate (#11360070; ThermoFisher Scientific) and 5% non-essential amino acids (#11140035; ThermoFisher Scientific) and kept in an incubator at 37 °C with 5% CO_2_. The transient transfections were performed with the calcium phosphate method as previously described [34]. For this, cells were plated on 6 well plates (#657160, Cellstar, Greiner, Germany) and grown for approximately 6 h to reach 80–90% confluence. 5 µg of expression plasmid each for TBX2 and its interaction candidate were co-transfected. Transfection efficiency was verified by epifluorescence of EGFP co-transfected with an empty *pcDNA3* vector.

We used the following expression vectors for transfections in HEK293 cells: *pcDNA3.huTBX2.HA* encoding N-terminally HA-tagged full-length human TBX2; *pCS2.Pbx1b* encoding full-length mouse PBX1B*,* (gift from Heike Pöpperl, Institute for Biophysical Chemistry, Hannover Medical School, Germany); *pd2EGFP-N1* (EGFP-expression vector) [35]; *pCMV6-Entry.Cbx3-Myc-DDK* encoding full-length mouse CBX3 (#MR224357, OriGene, Rockville, Maryland, USA); *pCMV-SPORT6.Hdac1* encoding full-length mouse HDAC1 (#4217199, Sourcebioscience, Nottingham, UK); *pCMV6-Entry.Hdac2-Myc-DDK* encoding full-length mouse HDAC2 (#MR226709, OriGene); *pCMV-SPORT6.Chd4* encoding full-length mouse CHD4 (#6489649, Sourcebioscience); *pCMV-Entry.Hmgb2-GFP* encoding full-length mouse HMGB2 (#MR202276, OriGene).

Cell lysates were obtained following the RIME protocol [26] as described for MS analysis. Immunoprecipitations were performed using primary antibodies against potential interaction partners of TBX2 either exploiting MYC protein tags (mouse anti-MYC monoclonal antibody (9E10), MA1-980, Thermo Fisher Scientific) or with antibodies directed against the respective protein (rabbit anti-HP1 gamma (CBX3) polyclonal antibody, #PA5-30954, ThermoFisher Scientific; mouse anti-CHD4 antibody [3F2/4], ab70469, Abcam; rabbit anti-HDAC1 polyclonal antibody, #PA1-860, ThermoFisher Scientific; rabbit anti-HDAC2 polyclonal antibody, #51-5100, ThermoFisher Scientific; rabbit anti-PBX1 polyclonal antibody, #PA5-82100, ThermoFisher Scientific). Antibodies for IP reactions were diluted according to manufacturers’ instructions. Cell lysates were incubated with respective antibodies for 1 h at room temperature, followed by incubation with ProteinG Magnetic Beads (#9006S, Cell Signaling Technology) overnight at 4 °C. After washing, beads were boiled in 1 × Laemmli buffer with 2.5% β-mercaptoethanol (CAS 60-24-2, Sigma Aldrich). Proteins were separated by SDS-PAGE and blotted onto PVDF membranes (T830.1, Carl Roth, Karlsruhe, Germany). Western blots were stained using HRP coupled mouse anti HA (#ab1265, Abcam) antibodies for detection of HA tagged TBX2. Bands were visualized using CheLuminate-HRP FemtoDetect chemiluminescent substrate (#A7807, AppliChem, Darmstadt, Germany).

### Documentation

Lung sections were documented with a DM5000 microscope (Leica Camera, Wetzlar, Germany) equipped with a Leica DFC300FX digital camera. Images were processed and analyzed with Adobe Photoshop CS5 (Adobe, San Jose, CA, USA) and ImageJ software (https://imagej.nih.gov). Western blots Blots were documented on a LAS-4000 luminescent Image Analyzer (Fuji, Tokyo, Japan).

## Results

### ChIP-Seq analysis identifies genome-wide TBX2 binding sites in the developing lung

**Fig. 1:**
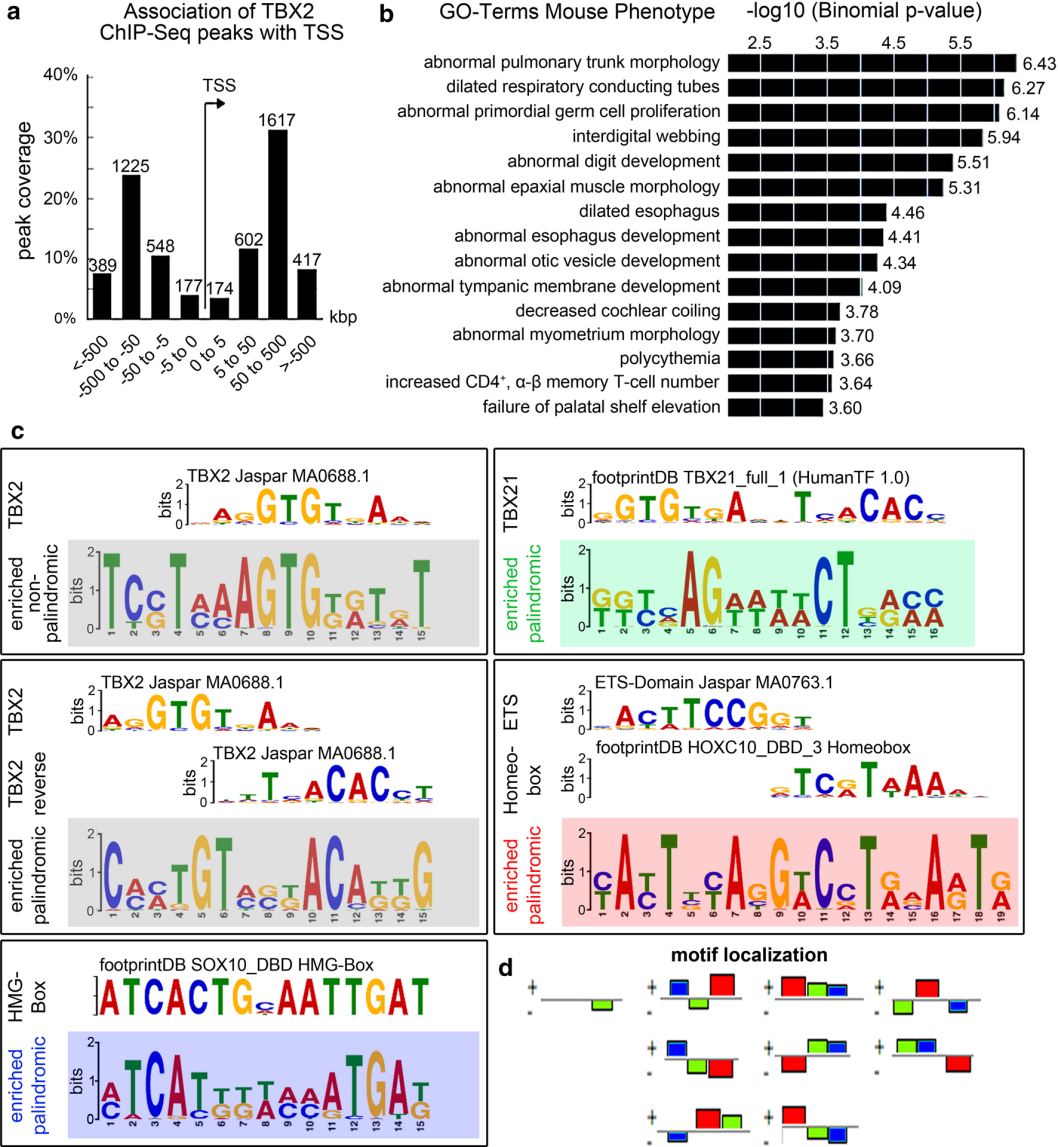
ChIP-Seq analysis identifies genomic binding sites of TBX2 in E14.5 lungs. **a**, **b** Analysis of TBX2 ChIP-sequencing peaks with Genomic Regions Enrichment of Annotations Tool (GREAT, version 4.0.4). **a** Bar diagram showing the orientation and distance of TBX2 ChIP peaks to a transcription start site (TSS). **b** Functional annotation shows enrichment of genes associated with TBX2 ChIP peaks in clusters with annotated mouse phenotypes and biological processes sorted by − log10 binomial p-value. **c** De-novo motif analysis was performed in Galaxy using FIMO—Scan a set of sequences for motifs (Galaxy v4.11.1.0) Novel consensus sequences are highlighted in colored boxes and compared to known motifs with TomTom Motif Comparison Tool v5.1.1. One palindromic and one non-palindromic motif with similarities to a known TBX2 binding element in the Jaspar database were discovered with E-values of 5.9e−198 and 4.6e−152 (grey boxes). Additional novel palindromic sequences show similarities to a TBX21 binding site in the footprint database, E = 1.7e−252 (green box), an ETS (Jaspar database) and homeobox (footprint database) binding motif, E = 6.8e−497 (red box), and an HMG-Box binding site (footprintDB), E = 4.4e−300 (blue box). **e** Analysis of motif localization by GREAT discovered conjunct motifs for TBX2 (green), ETS/homeobox (red) and HMG-box proteins (blue) in TBX2 ChIP-Seq peaks. Motifs are colored as in **c** and colored boxes in **d** reflect spatial arrangement and interconnection of motifs on both DNA strands (+ and −)

To obtain an unbiased view of TBX2-bound genomic regions in the pseudoglandular stage of lung development, we performed in vivo ChIP-Seq analysis on E14.5 wildtype lungs using an anti-TBX2 antibody. Mapping of sequenced tags using MACS2 callpeak [19] identified 3062 peaks that were at least 3.5 fold enriched with − log10 p-values between 4 and 256. Peak scores ranged from 7 to 2470 (Additional file 2: Table S1). We mapped TBX2 ChIP-sequencing peaks to genes with the Genomic Regions Enrichment of Annotations Tool (GREAT, version 4.0.4, http://great.stanford.edu/public/html) [36]. With respect to the transcription start site (TSS), 177 TBX2-binding sites mapped 5 kbp upstream, 174 mapped 5 kbp downstream; an additional 1150 TBX2-binding sites were located within 50 kbp up- or downstream; 3648 TBX2-binding sites were located at a greater distance (Fig. 1a). Since TBX2-binding sites can be associated with more than one gene, the number of total localizations does not sum up to the number of peaks found.

Gene ontology (GO) annotation of biological function and processes by GREAT revealed enrichment of peak-associated genes with various mouse phenotypes. ”Abnormal pulmonary trunk morphology” and “dilated respiratory conducting tubes” were the top enriched clusters in mouse phenotypes indicating significant affiliation of TBX2-bound regions to pulmonary development. Additional peak clusters were affiliated with the terms “abnormal digit development”, “failure of palatal shelf elevation”, “development of the urogenital system” and “limbs” reflecting known functions of TBX2 in mouse development [37–40]. “Abnormal otic vesicle development”, “decreased cochlear coiling” and “abnormal tympanic membrane morphology” within the top 15 clusters may indicate an as yet unexplored function associated with TBX2 expression in the otic vesicle [41] (Fig. 1b, Additional file 2: Tables S2–S4).

We next performed de novo sequence motif analysis on the sequenced tags with the FIMO tool in Galaxy [22] (Fig. 1c). Using the TomTom Motif Comparison Tool version 5.1.1 [23], we compared enriched motifs with experimentally determined transcription factor binding profiles deposited in Jaspar (http://jaspar.genereg.net) and footprintDB (http://floresta.eead.csic.es/footprintdb) databases. We found five enriched binding motifs in our ChIP-Seq data set with three strongly resembling previously described binding sites for T-box proteins. Two of them, one palindromic, the other non-palindromic, demonstrated high similarity to a known binding motif for TBX2 (entry MA0688.1 in Jaspar) (Fig. 1c, highlighted in grey); a third (palindromic) motif was highly similar to a TBX21 binding site (entry TBX21_full_1 in footprintDB HumanTF 1.0) (Fig. 1c, highlighted in green). The fourth motif matched a high mobility group (HMG)-box binding site (Fig. 1c, highlighted in blue), the fifth one resembled a composite of an erythroblast transformation specific (ETS) transcription factor binding site and a homeobox consensus sequence (Fig. 1c, highlighted in red). Strikingly, the TBX21-like binding motif occurred in different spatial combinations with the ETS-/homeobox- and HMG-motifs (Fig. 1d), raising the possibility of cooperative binding of TBX2 with transcription factors harboring the respective DNA binding domains.

### Microarray analysis identifies functional targets of TBX2 activity in the pulmonary mesenchyme

**Fig. 2:**
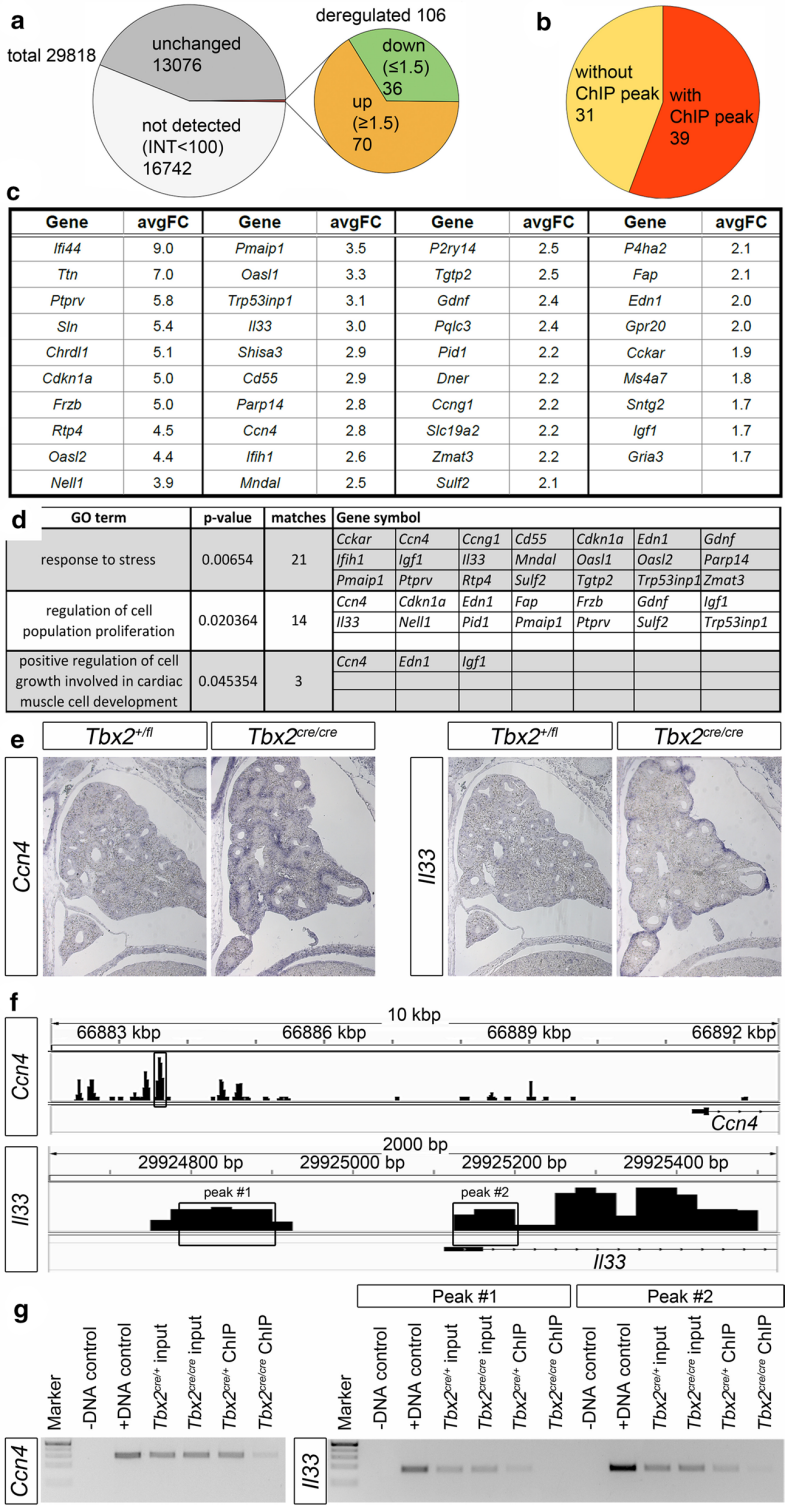
Microarray analysis identifies functional targets of TBX2 activity in E14.5 lungs. **a** Pie-chart summarizing the results of 4 individual transcriptional profiling experiments by microarrays of E14.5 control and *Tbx2*-deficient lungs. **b** Intersection of the list of genes upregulated in the microarrays of E14.5 *Tbx2-*deficient lungs and the list of genes associated with TBX2 ChIP peaks in the E14.5 lung. **c** List of genes upregulated in the microarrays of E14.5 *Tbx2*-deficient lungs and having a TBX2 ChIP-peak. Shown are the average fold changes (avgFC) of the 4 individual microarray data sets. **d** Functional annotation analysis by MouseMine websoftware identifies functional enrichment of terms related to stress response and growth control in the set of 39 genes upregulated in the microarrays of E14.5 *Tbx2*-deficient lungs and having a TBX2 ChIP-peak. **e** RNA in situ hybridization analysis of *Ccn4* and *Il33* expression on sections of E14.5 control and *Tbx2*-deficient lungs. **f** Scheme depicting the genomic loci of *Ccn4* and *Il33*. Binding peaks identified by ChIP-Seq analysis are indicated above. Black boxes indicate peaks further validated by ChIP-PCR. **g** ChIP-PCR-validation of peaks in *Il33* and *Ccn4* as indicated in (**f**). Lanes were loaded as indicated

ChIP provides genomic DNA fragments bound by TBX2 but does not necessarily reflect a biological functionality of near-by genes. To identify genes whose expression depends on TBX2 in lung development, we interrogated a microarray-based gene expression profiling data set previously generated from E14.5 lungs of *Tbx2*-deficient and control mice [13]. Filtering each of the four individual microarray data sets by thresholds for intensity (> 100) and fold change (> 1.4) delivered a set of 36 genes with reduced and a set of 70 genes with increased expression (Fig. 2a, Additional file 2: Tables S5, 6).

Since TBX2 is a potent transcriptional repressor [42–45], we intersected the list of upregulated genes with the list of genes with an associated TBX2 ChIP-peak, and obtained 39 genes that are potentially directly repressed by TBX2 in the developing lung (Fig. 2b, c). Functional annotation using MouseMine websoftware MGI 6.14 [25]) revealed an enrichment of clusters of GO terms related to “response to stress” (GO:0006950); “regulation of cell population proliferation” (GO:0042127) and “positive regulation of cell growth in cardiac muscle development” (GO:0061051) implicating TBX2 transcriptional activity in proliferative growth control (Fig. 2d, Additional file 2: Tables S7, 8). RNA in situ hybridization analysis on sections revealed a clear mesenchymal upregulation in *Tbx2*-deficient lungs for five genes: *Cdkn1a, Frzb1* and *Shisa3* as previously reported [13, 15], and additionally *Ccn4 (*also known as *Wisp1)* and *Il3*3 (Fig. 2e, Additional file 1: Figure S1). Analysis at earlier stages showed that derepression starts around E12.5 in *Tbx2*-deficient lungs (Additional file 1: Figure S2). Ectopic expression of *Il33* occurred in the mesothelium and the sub-mesothelial mesenchyme (Fig. 2e, Additional file 1: Figure S2).

To gain further evidence for a direct regulation of *Ccn4* and *Il33* by TBX2, we manually analyzed the ChIP-peak landscape for both genes (Fig. 2f). We detected peaks upstream of or within the promoter region that we evaluated by ChIP-PCR on wildtype and *Tbx2*-mutant lungs (Fig. 2g). Input control was comparable in wildtype and mutant chromatin for all tested peak regions. PCR signals in mutant chromatin were strongly reduced for all tested ChIP regions further implicating *Il33* and *Ccn4* as direct targets of TBX2 repressive activity in the pulmonary mesenchyme.

### Proteomic analysis identifies binding partners of TBX2 in the developing lung

**Fig. 3:**
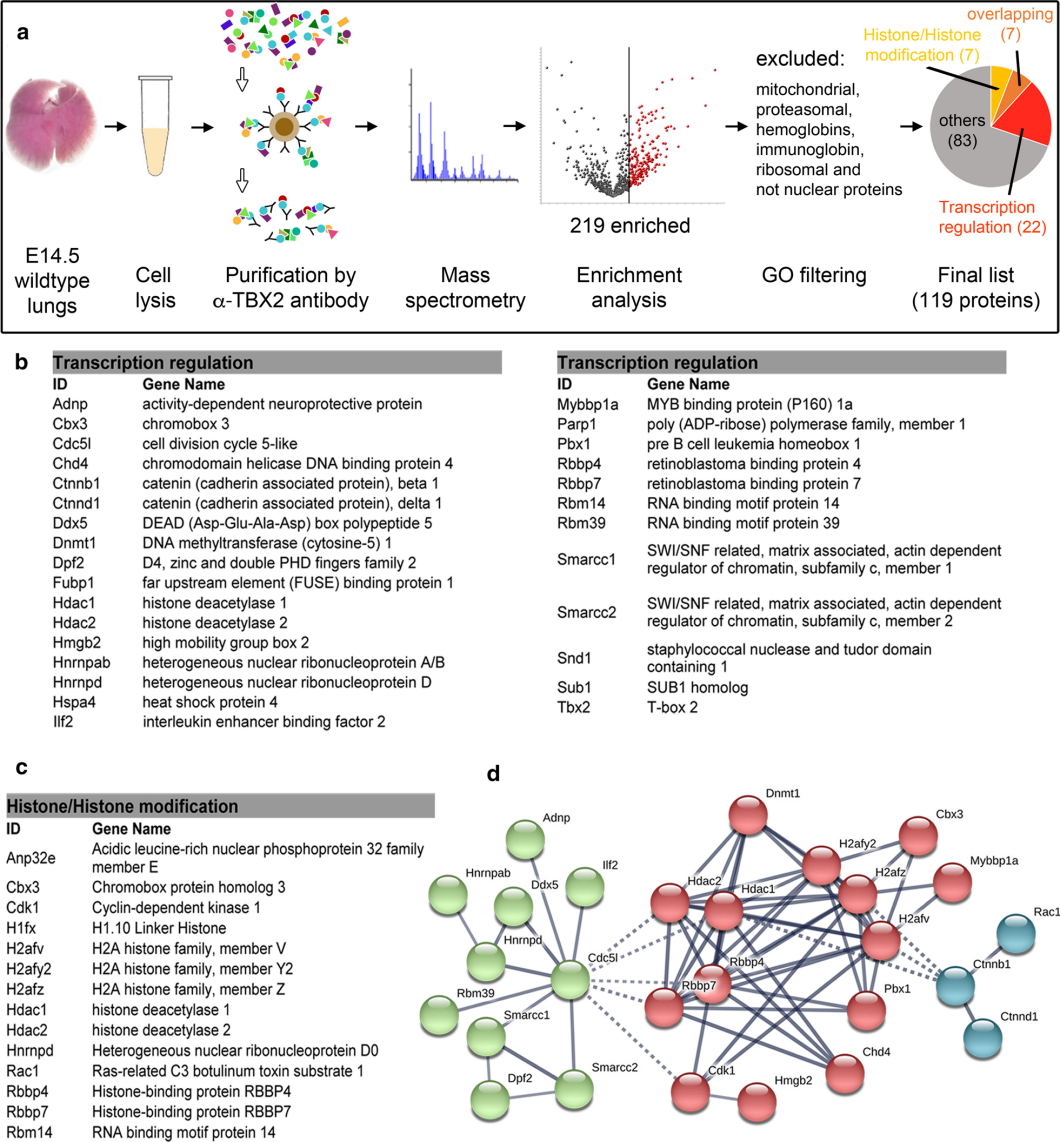
LC–MS/MS identifies TBX2 interaction partner in E14.5 lungs. **a** Diagram depicting the strategy to identify TBX2 interacting proteins in embryonic lungs. Tissue of E14.5 wildtype formaldehyde fixed lungs was homogenized, cells were lysed, and nuclei extracted. Protein complexes containing TBX2 were purified with an α-TBX2 antibody. Subsequent LC–MS/MS analysis and statistical filtering (Student's *t*-test difference of ≥ 2) revealed an enrichment of 219 proteins within the α-TBX2 fraction compared to the control lacking the α-TBX2 antibody. Manual exclusion of mitochondrial, proteasomal, and ribosomal proteins as well as hemoglobins, immunoglobins and non-nuclear proteins lead to a list of 119 candidate proteins. Of these, 22 were associated with the GO term “transcriptional regulation”, 7 with the terms “histone/histone modification”. 7 proteins were in the intersection of both GO term lists. **b**, **c** List of enriched proteins associated with the GO term “transcription regulation” (**b**) and “histones” or “histone modification” (**c**) according to DAVID functional analysis. **d** STRING analysis of interactions of the candidate proteins shown in (**b**) and (**c**). Three clusters were identified using MCL clustering with an inflation parameter of 2, an interaction score of high confidence (0.700) and deactivating the interaction source "textmining"

To identify protein interaction partners that may explain target specificity and transcriptional repressive activity of TBX2 in the pulmonary mesenchyme, we used an in vivo co-immunoprecipitation (Co-IP) approach from E14.5 lungs with subsequent liquid chromatography–tandem mass spectrometry analysis (LC–MS/MS) (Fig. 3a). For this, TBX2 containing complexes were purified from formaldehyde fixed lungs of E14.5 wildtype mice by affinity purification using an anti-TBX2 antibody coupled to Protein-G magnetic beads. The purified protein complexes of three independent experiments were sent to the proteomics facility of Hannover Medical School for protein extraction, and subsequently handed over to the Institute of Plant Genetics of Leibniz-University Hannover for LC–MS/MS analysis. In the three experiments, fragments of 919 mouse proteins were identified. An enrichment of 2 or larger (Student’s *t*-test) against the control (immunoprecipitates in absence of the anti-TBX2 primary antibody) was found for 219 proteins (Fig. 3a, Additional file 2: Table S9). We rejected hemoglobins, immunoglobins and proteins associated with the terms “ribosomal”, “mitochondrial” and “proteasomal” in the DAVID functional annotation tool (v6.8, david.ncifcrf.gov) reducing the list of candidates to 183 proteins. GO enrichment analysis using DAVID revealed that 119 of these proteins were associated with the term “nucleus”, i.e. were likely to colocalize with TBX2 in the nucleus (Fig. 3a, Additional file 2: Table S10). Out of this list, 29 proteins were annotated by DAVID with the GO term “regulation of transcription”, 14 proteins were associated with “histones or histone modification”, implicating a role in transcriptional regulation. Seven proteins were in common between the two lists: CBX3, HDAC1/2, HNRNPD, RBBP4/7 and RBM14 (Fig. 3a–c, Additional file 2: Table S10). Analysis of the protein association network of these 36 proteins using the STRING Protein–Protein Interaction Networks Functional Enrichment analysis tool (v11, https://string-db.org)) [30] uncovered three distinct protein interaction clusters (Fig. 3d). Within the largest cluster (in red in Fig. 3d) five proteins are known to be part of the transcriptional corepressor nucleosome remodeling and deacetylase (NuRD) core complex: the histone deacetylases HDAC1 and HDAC2, the histone-binding proteins RBBP4 and RBBP7, and the ATP-dependent chromatin-remodeling enzyme chromodomain-helicase-DNA-binding protein CHD4 [46, 47]. Proteins associated with this core complex included CBX3 (aka HP1γ), a chromatin organization modifier (Chromo) domain protein associated with heterochromatin [48], the homeobox transcription factor PBX1 that interacts with HOX proteins and is able to repress transcription [49], the HMG box containing protein HMGB2, which binds to DNA in a DNA structure-dependent but nucleotide sequence-independent manner to function in chromatin remodeling [50], the DNA (cytosine-5) methyltransferase DNMT1 that acts in gene silencing [51], and the transcriptional corepressor MYBBP1A [52].

The second cluster (green in Fig. 3d) contained several proteins implicated in RNA metabolism and splicing (HNRNs, DDX5, RBM39, CDC5L, ILF2). Further, members of the SWI/SNF chromatin remodeling complex were present (SMARCC1/2, DPF2). However, important core proteins of this complex including the ATPase (SMARCA2/4) were not enriched in our anti-TBX2 immunoprecipitation experiments. The third cluster (blue in Fig. 3d) represents a very small group of WNT-signaling associated proteins correlated with cell adhesion. For the two latter clusters interactions have been found only between individual components indicating lack of functional complex formation.

### TBX2 colocalizes and interacts with members of the NuRD complex (CHD4, HDAC1, HDAC2) as well as with PBX1, HMGB2 and CBX3

For further validation, we decided to employ candidate proteins found in the repressive NuRD complex (CHD4, HDAC1, HDAC2) as well as the proteins possibly associated with this complex (PBX1, HMGB2, CBX3) since they are likely to explain the target specificity and repressive activity of TBX2 in the pulmonary mesenchyme.

**Fig. 4:**
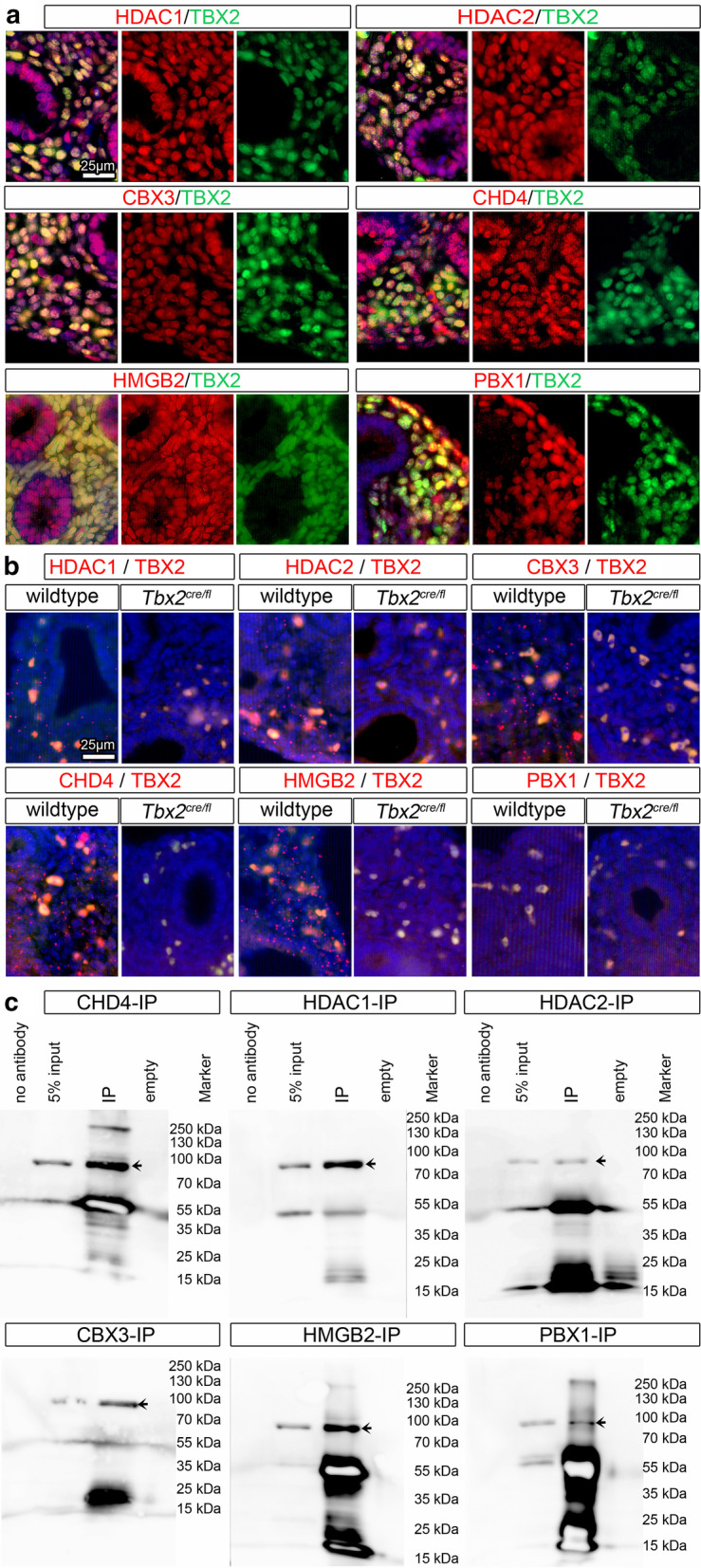
Interaction candidates are coexpressed with TBX2 in the pulmonary mesenchyme and interact in HEK293 cells. **a** Co-immunofluorescence analysis of candidate interaction partners (red) and TBX2 (green) on frontal sections of the right lung of E14.5 *Tbx2*^*cre/*+^ embryos. Antigens are color-coded and nuclei were counterstained with DAPI (blue). Insets or selected regions in overview images are magnified in rows 2,4 and 6. **b** In situ proximity ligation assay of TBX2 and candidate interaction partners on 10 µm frontal sections of E14.5 wildtype and *Tbx2*^*cre/fl*^ mutant lungs. Direct interaction is visualized by small red fluorescent dots. Larger more diffuse orange stains are due to auto-fluorescence of blood cells. Nuclei are counterstained with DAPI (blue). **c** Western blot analysis of co-immunoprecipitation experiments for verification of TBX2 interaction with candidate proteins on 10% SDS polyacrylamide gels. Detection was performed with an anti-TBX2 primary antibody and developed with chemoluminescence-IHC. Arrows indicate TBX2 bands. Lanes were loaded as follows: No antibody: IP without specific antibody resembling negative IP-control; 5% input: 5% of crude cell extract before precipitation; empty: no protein loaded; IP: co-immunoprecipitate with antibody for specific candidate. Expected molecular weight for TBX2.HA approx. 76.2 kDa

Co-immunofluorescence analysis of the candidate proteins and TBX2 on transverse sections of E14.5 lungs revealed that all six candidates were widely coexpressed with TBX2 in the nuclei of pulmonary mesenchymal cells (Fig. 4a).

The proximity ligation assay [32, 33] detected strong nuclear signals in mesenchymal cells on sections of wildtype but not of *Tbx2*-mutant lungs for combinations of TBX2 with CHD4, HDAC1, HDAC2, HMGB2 and CBX3 but not for PBX1, indicating specific and direct interaction in short distance with most of our candidates (Fig. 4b).

**Fig. 5:**
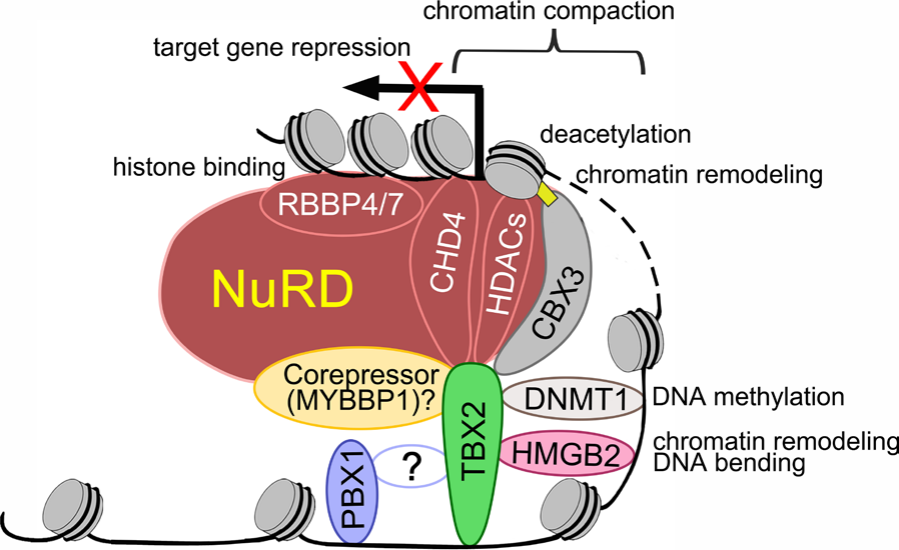
Scheme of the molecular interactions of TBX2 in repression of target genes in the pulmonary mesenchyme. Grey discs represent nucleosomes. DNA is displayed as black line. Dotted line indicates unknown length of DNA fragment and possible long distance. Circles for proteins do not represent the real size. TBX2 binds in concert with HMGB2 and PBX1 to specific DNA sites in enhancer regions. Binding to PBX1 is probably indirect via unknown proteins, binding to HMGB2 is direct and may aid in chromatin bending and bringing enhancer regions to the proximity of transcription start sites. TBX2/DNMT1 binding may induce DNA methylation to repress gene expression. TBX2 and CBX3 directly bind and both associate with the NuRD complex for chromatin remodeling. TBX2/MYBBP1 interaction may strengthen the association with the NuRD complex to repress target genes. TBX2 directly interacts with components of the NuRD complex, namely HDACs for chromatin deacetylation and the chromatin remodeling complex CHD4. TBX2 interaction with CBX3, HDACs and CHD4 collectively induces chromatin compaction. RBBP4/7 binds to chromatin to consolidate binding of the NuRD-complex to target gene DNA

In co-transfection/co-immunoprecipitation experiments in HEK293 cells, TBX2 interacted with all six candidates (Fig. 4c). Hence, TBX2 interacts in the mesenchymal compartment of the developing lung with proteins implicated in transcriptional repression (Fig. 5).

## Discussion

### *Il33* and *Ccn4* are novel direct targets of TBX2 in the lung mesenchyme

We previously performed a ChIP-Seq experiment to validate *Cdkn1a*, *Cdkn1b, Frzb* and *Shisa3* as direct targets of TBX2 repressive activity in the pulmonary mesenchyme [13]. Here, we performed a new ChIP-Seq experiment to survey in an unbiased fashion the genomic binding sites of TBX2 in this organ. Importantly, we increased the chromatin input to obtain higher signals and performed bioinformatical analysis on the obtained called peak data set. We identified 3062 significantly enriched binding sites in the mouse genome that were variably spaced from TSSs indicating distant enhancer-promoter interactions. By a number of criteria, we deem that these binding peaks represent or at least contain *bona fide* TBX2 genomic binding sites. First, our motif analysis found a highly significant enrichment of DNA sequences similar to a T-box binding element initially identified in an in vitro binding site selection approach for the prototypical T-box protein Brachyury and to a consensus sequence previously identified by ChIP-Seq for TBX2 in neuroblastoma cell lines [53, 54]. Second, we recovered binding peaks in those genes previously characterized as direct targets of TBX2 repressive activity in the lung, including *Cdkn1a*, *Shisa3* and *Frzb* [13, 15]. Third, GO annotation of biological function and processes revealed enrichment of peak-associated genes with mouse phenotypes previously associated with TBX2 function in various embryological contexts [37–40].

The intersection of transcriptional profiling and ChIP-seq data sets provided a list of 39 genes that might be directly regulated by TBX2. In line with our previous phenotypic characterization, we found enrichment of genes annotated with proliferation and stress control, indicating that TBX2 predominantly represses anti-proliferative genes. To our surprise, we failed to detect increased expression of most candidate genes in the pulmonary mesenchyme of *Tbx2*-deficient embryos by in situ hybridization analysis. We assume that the overall expression of these genes is too low in the pulmonary mesenchyme of *Tbx2*-deficient embryos to reliably detect it by this method. Since many of these candidate genes are strongly expressed in the epithelium, changes in the mesenchyme are unlikely to be detected either by alternative approaches including RT-PCRs of whole lung tissue. However, we confirmed increased expression of *Ccn4* and *Il33* in the lung mesenchyme of mutant embryos, and validated them as additional direct targets of TBX2 by ChIP-PCR. CCN4, also known as WISP-1, is a member of the WNT1 inducible signaling pathway protein (WISP) subfamily of the connective tissue growth factor/CCN family of matricellular proteins. CCN proteins, which are secreted, interact with cell surface receptors (e.g., integrins) and extracellular matrix components to modulate cellular functions. CCN4 can stimulate proliferation, adhesion, invasion, metastasis and epithelial-to-mesenchymal transition of cells [55]. The significance of repression of *Ccn4*, and thus, of these cell programs in the lung mesenchyme cannot be answered at this point. *Il33* codes for a cytokine which mediates inflammatory responses [56]. Its repression by TBX2 in the mesothelium and the submesothelial mesenchyme might prevent a premature activation of these responses in lung development, and thus avoid excessive immune cell infiltration at this stage.

### TBX2 interacts with homeobox and HMG-box transcription factors in the lung mesenchyme

Our de novo motif analysis of the TBX2-ChIP-seq data set did not only reveal binding sites highly similar to the consensus binding site(s) of the T-box DNA-binding domain [11, 53] but also in variable spatial association for homeobox-, ETS-domain and HMG-box proteins, indicating concerted or even cooperative DNA-binding of TBX2 with members of other transcription factor families. Since DNA-binding sites are normally rather short, concerted binding of several transcription factors to adjacent binding sites dramatically increases target specificity [57]. It may further enhance the transcriptional outcome and may serve architectural purposes. In fact, high-mobility group (HMG) proteins are architectural DNA bending proteins that promote DNA loop structures and tether distant regulatory elements to gene promoters [58].

Most satisfyingly, we identified the homeobox transcription factor PBX1 and the HMG-box protein HMGB2 that have both been implicated in transcriptional repression [59, 60], amongst TBX2 interaction partners in our unbiased proteomic screen in the E14.5 lung. We validated binding of these candidates to TBX2 in co-immunoprecipitations in HEK cells, and showed that they are largely coexpressed with TBX2 in the lung mesenchyme at E14.5. Interestingly, only HMGB2 interacted with TBX2 in the proximity ligation assay, suggesting PBX1 binding to adjacent DNA sites without direct physical interaction with TBX2 in vivo. Mice with loss of *Hmgb2* do not exhibit lung defects, while *Pbx1*-deficiency results in lung hypoplasia and alveolar defects [61, 62]. In either case it is conceivable that the interaction with TBX2 is irrelevant for mesenchymal proliferation and branching morphogenesis in the pseudoglandular stage. Alternatively, redundancy with closely related family members (*Hmgb1* and *Pbx2-4*) may conceal the requirement of these genes in these cellular programs.

Although our de novo motif analysis found an enrichment of an ETS-domain binding motif in the TBX2-ChIP peaks, we did not identify a member of this protein family in our proteomic screen. This seems plausible since members of the ETS transcription factor family (e.g. ETV4, ERG, ELF1, ELK1) act as transcriptional activators [63–65] and would interfere with the repressive activity of TBX2, PBX and HMGB2 complexes. However, localization of these motifs might not occur coincidentally. It is conceivable that TBX2 inhibits ETS-mediated transcriptional activation competitively or by displacement of ETS transcription factor complexes from the promoter without necessarily interacting directly.

It is important to note that interaction of TBX2 and the closely related TBX3 with HMG-box and homeobox proteins has been documented before for other developmental contexts in which these closely related T-box proteins act [66–68] while interaction with ETS domain proteins is unreported. This further substantiates the notion that TBX2 preferentially interacts with HMG and possibly homeobox proteins in target gene repression in the lung.

### TBX2 interacts with the components and interaction partners of the repressive NuRD complex

It is long known that TBX2 acts as repressor of target gene transcription both in vitro and in vivo [42–45] but evidence has accumulated that the molecular mechanisms of repression may differ in different developmental contexts. In the developing heart, TBX2 achieves repression of chamber specific genes in the atrioventricular canal by competing with the transcriptional activator TBX5 for binding to both conserved T-box binding elements as well as cooperating transcription factors including NKX2-5 and GATA4 [66]. In breast cancer cell lines, TBX2 interacts with EGR1 to co-repress EGR1-target genes including the breast tumor suppressor gene *NDRG1*. To do so, TBX2 recruits the DNA methyltransferase DNMT3B and histone methyltransferase complex components to set a repressive chromatin mark (H3K9me3) within the proximal promoter of *NDRG1* [69]. In contrast, the repression of *Cdkn1a, Cdkn2a*, *Adam10*, *Pten* and muscle-specific genes in different cancer cell lines or myoblasts cells depends on recruitment of HDAC1, hence, deacetylation of lysine residues in N-terminal tails of histones [70–73]. The closely related T-box factor TBX3 also binds to HDACs (1,2,3 and 5) to repress target genes including *Cdkn1a* and *Cdkn2a* [74, 75].

Our proteomic analysis argues that HDACs namely, HDAC1 and HDAC2, are also involved in repression of TBX2 target genes including *Cdkn1a* and *Cdkn1b* in the lung mesenchyme. The two proteins were enriched in our proteomic screen, they bound to TBX2 in HEK cells, and they were found in short distance, direct association with TBX2 in nuclei of lung mesenchymal cells. Our proteomic analysis further identified RBBP4, RBBP7 and CHD4 which are known to interact with HDAC1 and HDAC2 in the CHD/NuRD complex [46, 47] implicating for the first time this chromatin remodeling/histone deacetylase complex in the repression of TBX2 targets genes in the lung mesenchyme.

CHD proteins like CHD4 are known to bind to methylated histone tails (H3K9me3) most likely via their PHD2 finger [76]. Similarly, CBX3 (aka HP1γ), another protein for which we confirmed TBX2 binding, recognizes H3K9me3 marks and is involved in heterochromatin formation and transcriptional silencing including that of *Cdkn1a* by TBX2 [48, 69, 77, 78]. Together, this would argue for TBX2 interaction with histone-methyltransferases such as was shown for repression of *NDRG1* in tumor cells [69]. The identity of such histone methyltransferases in the lung mesenchyme remains open since we did not detect such enzymes in our proteomic screen. However, similar to the control of *NDRG1* by TBX2, we found that a DNA methyltransferase, namely (maintenance) DNMT1 coprecipitated with TBX2 from lung tissue implicating DNA methylation in transcriptional repression by TBX2 [51]. Intriguingly, cooperation of DNMTs with HDACs and the NuRD complex, and of DNMTs with CBX3/HP1γ and the NuRD complex in gene silencing including that of sFRPs (such as *Frzb*) and of *Cdkn1a* has been reported, substantiating the relevance of TBX2 interaction with these components [79–82].

We also found MYBBP1A as an interaction partner in the proteomic analysis. MYBBP1A acts as a corepressor for different transcription factors and is possibly involved in chromatin compaction by recruiting negative epigenetic modifiers, such as HDAC1/2 and histone methyltransferase [52, 83]. Finally, in the group of 119 enriched nuclear proteins LMNB1 was present, localizing TBX2 targets to the heterochromatic region associated with the nuclear lamina.

### TBX2-NuRD interaction: a possible target for anti-cancer therapy

HDACs have an important role in regulation of gene expression. The removal of acetyl groups from lysine residues at histone tails leads to chromatin compaction, which inhibits the binding of transcription machinery at the promoter region, thus, represses gene transcription. Expression of many HDACs is increased in malignant cells, and HDACs have been linked with acquisition of malignant phenotypes in cancerogenesis by stimulation of proliferation, cell survival and motility, inhibition of differentiation and promotion of angiogenesis [84, 85]. Similarly, the NuRD complex has been implicated in the regulation of transcriptional events that are integral to oncogenesis and cancer progression [86].

Increased proliferation in tumor cells frequently correlates with reduced expression levels of cyclin-dependent kinase inhibitors, including CDKN1A. Transcription of *Cdkn1a* is significantly decreased by the upregulation of HDACs and the NuRD complex whereas transient overexpression of HDACs induce *Cdkn1a* repression [87, 88]. For these reasons inhibitors of HDACs have been highlighted as a novel category of anti-cancer drugs. However, due to the widespread involvement in many transcriptional programs in healthy cells, inhibition of HDACs is likely to have severe side effects in cancer therapy [89, 90].

HDACs are recruited to specific gene loci by tissue-specific transcription factors. Our work has characterized TBX2 as a factor that recruits HDAC1,2 and the NuRD complex to anti-proliferative target genes including *Cdkn1a* in embryonic lung mesenchyme. Interestingly, *TBX2* and the closely related *TBX3* gene are reactivated and/or overexpressed in several cancers including small cell lung carcinoma melanoma, breast, pancreatic, liver, and bladder cancers [91]. Moreover, TBX2 was characterized as a potent immortalizing factor that represses *Cdkn2a* and *Cdkn1a* to bypass senescence [44, 70]. It is likely that TBX2 and/or TBX3 account for the function of HDACs in repression of cyclin-dependent kinase inhibitor gene expression in at least some cancer types, and that TBX2 or of its specific interaction with HDACs represent valuable highly specific targets for anti-cancer therapy.

## Conclusion

Our work identified *Il33* and C*cn4* as additional direct target genes of TBX2 in the lung mesenchyme. It revealed combinations of T-box binding elements with bindings sites for HMG-box and homeobox proteins in the TBX2 genomic binding peaks, and characterized the transcription factors PBX1 and HMGB2, and components and interaction partners of the NuRD complex as TBX2 protein binding partners. We suggest TBX2 cooperates with homeobox and HMG-box transcription factors in transcriptional repression of anti-proliferative genes in the lung mesenchyme, and that this repressive activity relies on histone deacetylation and chromatin remodeling mediated by the NuRD complex but also on DNA methylation, histone H3K9 trimethylation and subsequent heterochromatin formation by CBX3 at the nuclear lamina (Fig. 5). Our findings may have implications for the understanding and treatment of tumors in which TBX2 is overexpressed.

## Supplementary Information

**Additional file 1: Figure S1.** Expression analysis of candidate genes with increased expression in microarray analyses of *Tbx2*-deficient lungs. **Figure S2.** Derepression of TBX2 target genes occurs around E12.5 in *Tbx2*-deficient pulmonary mesenchyme.

**Additional file 2: Table S1.** TBX2 ChIP-seq peaks. **Table S2.** Functional annotation of enriched TBX2 ChIP-seq peaks. **Table S3.** Functional annotation of enriched TBX2 ChIP-seq peaks. **Table S4.** Functional annotation of enriched TBX2 ChIP-seq peaks. **Table S5.** Genes with decreased expression in the microarrays of E14.5 control vs *Tbx2*-deficient lungs. **Table S6.** Genes with increased expression in the microarrays of E14.5 control vs *Tbx2*-deficient lungs. **Table S7.** GO-Term analysis of upregulated genes in microarray analysis. **Table S8.** Functional annotation analysis of upregulated genes in microarray analysis. **Table S9.** Mass Spectrometry. **Table S10.** GO-Term analysis of interaction candidates.

## Data Availability

All datasets and reagents are available from the corresponding author on reasonable request.

## Abbreviations

Å: Ångström
α-: Anti-
ADAM10: A disintegrin and metallopeptidase domain 10
ATP: Adenosintriphosphat
avgFC: Average fold change
BMP4: Bone morphogenetic protein 4
°C: Degree celsius
CBX3: Chromobox 3
CCN4: Cellular communication network factor 4
CDC5L: Cell division cycle 5-like
CDKN1A: Cyclin-dependent kinase inhibitor 1A (P21)
CDKN1B: Cyclin-dependent kinase inhibitor 1B (P27)
CHD: Chromodomain helicase DNA binding protein
CHD4: Chromodomain helicase DNA binding protein 4
ChIP: Chromatin immunoprecipitation
ChIP-Seq: Chromatin immunoprecipitation sequencing
Chromo: Chromatin organization modifier
cm: Centimeter
CO_2_: Carbon dioxide
Co-IP: Co-immunoprecipitation
DAPI: 4′,6-Diamidino-2-phenylindole
DDX5: DEAD box helicase 5
DNA: Deoxyribonucleic acid
DNMTs: DNA methyltransferases
DNMT1: DNA (cytosine-5) methyltransferase 1
DNMT3B: DNA methyltransferase 3B
DMEM: Dulbecco's Modified Eagle's Medium
DPF2: D4, zinc and double PHD fingers family 2
E: Embryonic day
EGFP: Enhanced green fluorescent protein
EGR1: Early growth response 1
ELF1: E74-like factor 1
ELK1: ELK1, member of ETS oncogene family
ERG: ETS transcription factor
ETS: Erythroblast transformation specific
ETV4: Ets variant 4
FC: Fold change
FCS: Fetal calf serum
Frzb: Fizzled-related protein
GATA4: GATA binding protein 4
GO: Gene ontology
GREAT: Genomic Regions Enrichment of Annotations Tool
H_2_O_2_: Hydrogen peroxide
HDACs: Histone deacetylases
HDAC1: Histone deacetylase 1
HDAC2: Histone deacetylase 2
HEK293 cells: Human embryonic kidney 293 cells
HMG: High mobility group
HMGB2: High mobility group box 2
HNRNs: Heterogeneous nuclear ribonucleoproteins
HNRNPD: Heterogeneous nuclear ribonucleoprotein D
HOX: Homeobox
HP1γ: Heterochromatin protein 1, gamma
hr: Hour
IgG: Immunglobulin G
IL33: Interleukin 33
ILF2: Interleukin enhancer binding factor 2
IP: Immunoprecipitation
kbp: Kilo base pairs
kDa: Kilodalton
kV: Kilovolt
LC–MS/MS: Liquid chromatography tandem mass spectrometry
LMNB1: Lamin B1
min: Minute
µg: Microgram
µl: Microliter
µm: Micrometer
mM: Millimolar
MS: Mass spectrometry
MYBBP1A: MYB binding protein (P160) 1a
NDRG1: *N*-myc downstream regulated gene 1
NKX2-5: NK2 homeobox 5
nl: Nanoliter
NuRD: Nucleosome remodeling and deacetylase
PBS: Phosphate-buffered saline
PBX1: Pre B cell leukemia homeobox 1
PCR: Polymerase chain reaction
PFA: Paraformaldehyde
PHD2: Plant homeodomain
PTEN: Phosphatase and tensin homolog
RBBP4: Retinoblastoma binding protein 4, chromatin remodeling factor
RBBP7: Retinoblastoma binding protein 7, chromatin remodeling factor
RBM14: RNA binding motif protein 14
RBM39: RNA binding motif protein 39
RNA: Ribonucleic acid
RT-PCR: Reverse transcription polymerase chain reaction
SDS: Sodium dodecyl sulfate
sFRPs: Secreted frizzled-related protein
SHISA3: Shisa family member 3
SMARCC1: SWI/SNF related, matrix associated, actin dependent regulator of chromatin, subfamily c, member 1
SMARCC2: SWI/SNF related, matrix associated, actin dependent regulator of chromatin, subfamily c, member 2
SMARCA2: SWI/SNF related, matrix associated, actin dependent regulator of chromatin, subfamily a, member 2
SMARCA4: SWI/SNF related, matrix associated, actin dependent regulator of chromatin, subfamily a, member 4
SMC: Smooth muscle cell
SWI/SNF: SWItch/Sucrose Non-Fermentable
TBX2: T-box 2
TBX21: T-box 21
TBX3: T-box 3
TBX5: T-box 5
TFA: Trifluoroacetic acid
TGFs: Transforming Growth Factors
TSA: Tyramide signal amplification
TSS: Transcription start site
v/v: Volume percent
WISP: WNT1 inducible signaling pathway protein
WISP-1: WNT1 inducible signaling pathway protein 1
WNT: Wingless-type MMTV integration site family
WNT1: Wingless-type MMTV integration site family, member 1
