# Supplementary material for: Combined genomic and proteomic approaches reveal DNA binding sites and interaction partners of TBX2 in the developing lung
Source: Respir Res. 2021 Mar 17;22:85. doi: 10.1186/s12931-021-01679-y (PMC7968368; doi:10.1186/s12931-021-01679-y)
Supplement: Supplementary file 1 — Additional file 1: Figure S1. Expression analysis of candidate genes with increased expression in microarray analyses of Tbx2-deficient lungs. Figure S2. Derepression of TBX2 target genes occurs around E12.5 in Tbx2-deficient pulmonary mesenchyme. [file 12931_2021_1679_MOESM1_ESM.docx]

**Additional file 1**


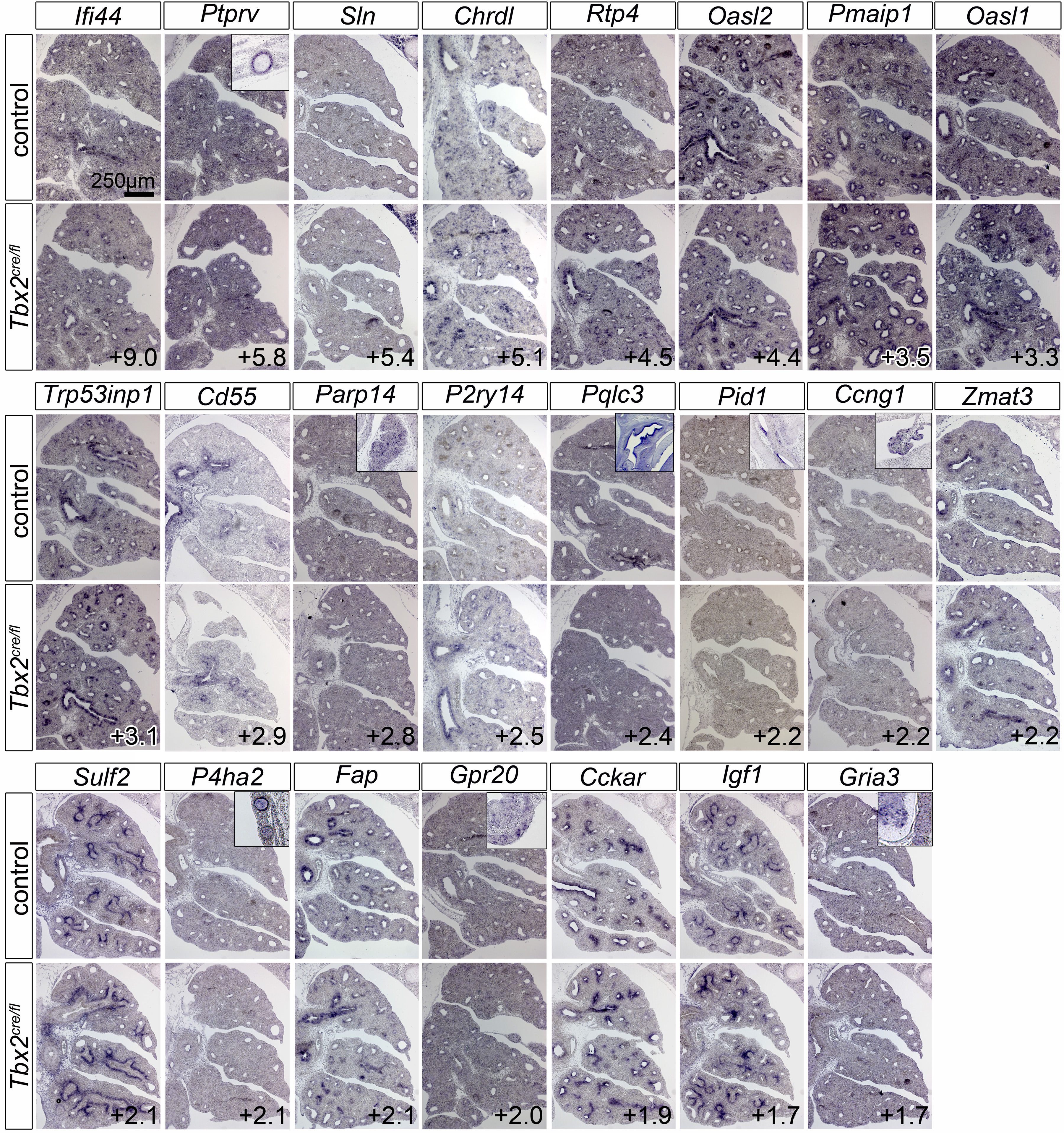


**Figure S1. Expression analysis of candidate genes with increased expression in microarray analyses of TBX2-deficient lungs.**

RNA i*n situ* hybridizations were performed on frontal lung sections of E14.5 control (*Tbx2^+/fl^*) and *Tbx2*-deficient (*Tbx2^cre/fl^)* embryos. Insets show positive control regions. Numbers refer to fold change in the microarray analysis of *Tbx2*-deficient lungs. Probes and genotypes are as indicated.

**
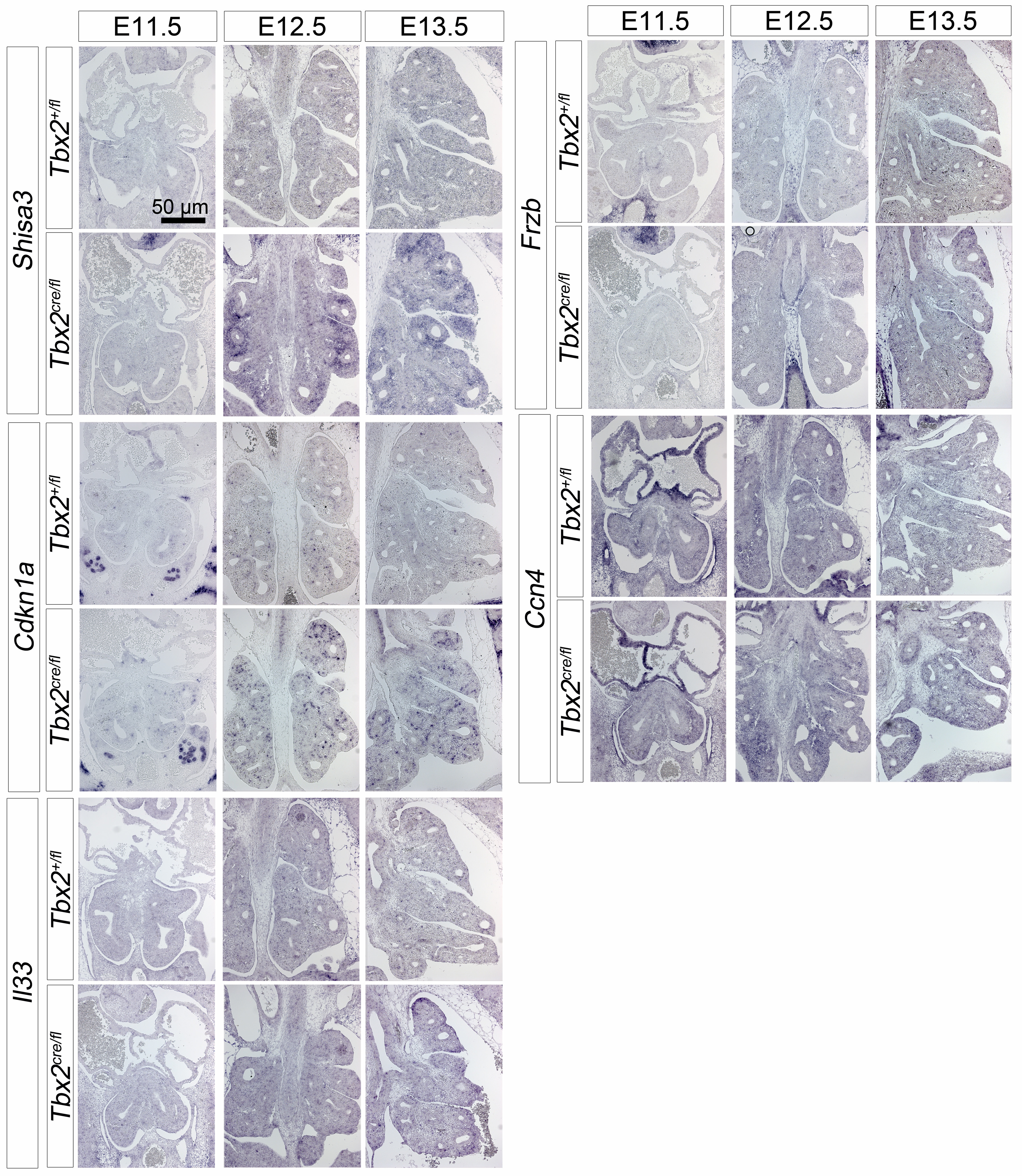
**

**Figure S2. Derepression of TBX2 target genes occurs around E12.5 in *Tbx2*-deficient pulmonary mesenchyme.**

*In situ* hybridization on frontal lung sections of E11.5, E12.5 and E13.5 control (*Tbx2^+/fl^*) and *Tbx2*-deficient (*Tbx2^cre/fl^)* mice. Probes, genotypes and stages are as indicated.
